# Advances in Mechanisms and Management Strategies of Pulmonary Hypertension: A Review

**DOI:** 10.31083/RCM49075

**Published:** 2026-10-14

**Authors:** Anqi Liu, Linfeng Xi, Jie Du, Yanlin Guo, Hanchi Yu, Shuai Zhang, Wanmu Xie, Min Liu

**Affiliations:** ^1^Chinese Academy of Medical Sciences & Peking Union Medical College, 100730 Beijing, China; ^2^Department of Radiology, China-Japan Friendship Hospital, 100029 Beijing, China; ^3^Department of Pulmonary and Critical Care Medicine, China-Japan Friendship Hospital; National Center for Respiratory Medicine; Institute of Respiratory Medicine, Chinese Academy of Medical Sciences; National Clinical Research Center for Respiratory Diseases, 100029 Beijing, China; ^4^State Key Laboratory of Respiratory Health and Multimorbidity, China-Japan Friendship Hospital, 100029 Beijing, China; ^5^Peking University Health Science Center, 100191 Beijing, China

**Keywords:** pulmonary hypertension, pathophysiology, disease management, pulmonary arterial hypertension

## Abstract

Pulmonary hypertension (PH) encompasses a heterogeneous group of disorders characterized by persistently elevated pulmonary artery pressure and driven by diverse etiologies and intersecting pathological mechanisms. In recent years, substantial advances have clarified key contributors to PH pathogenesis, including pulmonary vascular remodeling, immune–inflammatory dysregulation, and genetic predispositions. Concurrently, diagnostic and therapeutic approaches tailored to different PH subtypes have evolved, incorporating targeted pharmacological treatments, interventional procedures, and lung transplantation. This review summarizes recent advances in elucidating the mechanisms underlying PH and outlines clinical management strategies across the major etiologic categories. Particular emphasis is placed on developments in PH-specific therapies and on emerging therapeutic targets to provide a coherent mechanistic framework and practical guidance for clinicians involved in the diagnosis and treatment of this challenging disease.

## 1. Introduction

Pulmonary hypertension (PH) is a complex and multifaceted clinical syndrome characterized by elevated pulmonary artery pressure, which ultimately leads to right ventricular dysfunction, heart failure, and increased mortality. The hemodynamic definition of PH has evolved over time. The 2018 World Symposium on Pulmonary Hypertension revised the diagnostic threshold to a mean pulmonary artery pressure (mPAP) greater than 20 mmHg [1], and this definition has been formally adopted in the 2022 ESC/ERS guidelines [2]. PH represents a significant global health burden, affecting at least 1% of the world’s population, and a disproportionately higher impact in low- and middle-income countries (LMICs) [3]. According to the 2022 ESC/ERS classification, PH is categorized into five groups based on etiology, pathophysiology, and therapeutic approach: Group 1 includes pulmonary arterial hypertension (PAH), characterized by pulmonary vascular remodeling and increased pulmonary vascular resistance (PVR); Group 2 comprises PH due to left heart disease; Group 3 encompasses PH associated with lung diseases and/or hypoxia; Group 4 consists of PH associated with chronic pulmonary artery obstruction; and Group 5 includes PH with unclear or multifactorial mechanisms [2]. This classification facilitates etiologic diagnosis and guides management strategies.

Early diagnosis of PH remains challenging due to nonspecific symptoms and relies on a combination of non-invasive and invasive assessments, with right heart catheterization (RHC) remaining the gold standard [2]. Therapeutic management has advanced considerably, especially in PAH, where targeted pharmacotherapies have improved survival and the quality of life. In addition, interventional procedures, including pulmonary endarterectomy for chronic thromboembolic pulmonary hypertension (CTEPH) and balloon pulmonary angioplasty for inoperable cases, have expanded the therapeutic armamentarium [4,5]. Despite these advances, unmet needs persist, especially in non-PAH PH groups and in patients with comorbidities or advanced disease. The heterogeneity of PH underscores the need for more personalized approaches that integrate genetic, molecular, and hemodynamic profiling in order to optimize diagnosis and treatment [6,7]. Ongoing research into pathophysiological mechanisms, biomarkers, and novel therapies holds promise for enhancing the management and prognosis of this challenging condition. In this review, we summarize recent advances in the pathophysiological mechanisms, classification, diagnosis, and management of PH, while discussing current challenges and emerging therapeutic approaches.

## 2. Pathophysiological Mechanisms of Pulmonary Hypertension

### 2.1 Pulmonary Vascular Remodeling

Pulmonary vascular remodeling is the hallmark pathological endpoint of PAH, representing the structural manifestation of upstream genetic susceptibilities and immune dysregulation. The process is characterized by complex structural alterations involving all three layers of the pulmonary artery wall: the intima, media, and adventitia. The intimal layer thickens primarily due to endothelial cell dysfunction and proliferation, while the medial layer exhibits hypertrophy and hyperplasia of pulmonary artery smooth muscle cells (PASMCs). Adventitial remodeling is marked by fibroblast activation and extracellular matrix (ECM) accumulation, contributing to vessel wall stiffening and loss of compliance [8]. These structural changes collectively narrow the vascular lumen, increase PVR, and elevate pulmonary artery pressure, thereby increasing afterload on the right ventricle.

At the cellular level, pulmonary artery endothelial cells (PAECs) demonstrate abnormal proliferation and impaired apoptosis, leading to endothelial dysfunction and disruption of cellular integrity. This dysfunction is accompanied by phenotypic shifts, such as endothelial-to-mesenchymal transition (EndoMT), which further contributes to vascular remodeling and fibrosis [9,10]. PASMCs exhibit a phenotypic switch from a contractile to a synthetic state, characterized by enhanced proliferation, migration, and resistance to apoptosis, which results in medial hypertrophy and neointimal formation [11,12]. Adventitial fibroblasts (PAFs) also actively participate by proliferating and secreting ECM proteins, which remodel the adventitia and influence medial and intimal layers through paracrine signaling [8]. ECM remodeling is a critical component of pulmonary vascular structural changes. Alterations in ECM composition, including increased deposition of collagen, elastin, and proteoglycans, lead to vascular stiffening and reduced compliance. The basement membrane, a specialized ECM structure closely associated with endothelial and smooth muscle cells, undergoes dynamic remodeling affecting cell adhesion, migration, and signaling. Dysregulation of ECM-degrading enzymes such as matrix metalloproteinases (MMPs) and cross-linking enzymes like lysyl oxidase (LOX) contributes to pathological ECM accumulation and disorganization [13,14].

Rather than occurring in isolation, these ECM changes provide a scaffold for cellular proliferation and modulate mechano-transduction pathways that exacerbate vascular remodeling. Inflammatory processes and oxidative stress serve as critical amplifiers, inducing phenotypic changes in vascular cells and ECM alterations. Hypoxia, a common stimulus in PH, triggers signaling pathways that enhance PASMC proliferation and ECM production, including activation of calcium-sensing receptors and angiotensin-(1-7) pathways, which have been shown to modulate remodeling [15,16,17]. Parallel alterations in connexin 43 (Cx43) expression further distort vascular reactivity and remodeling, with nicotine exposure exacerbating these effects [18]. A major unresolved question in PAH pathogenesis is why the damaged endothelium fails to undergo adaptive regeneration, instead triggering a maladaptive, hyperproliferative cascade, a phenomenon that points toward a “second hit” driven by inflammatory crosstalk.

### 2.2 Inflammation and Immune Mechanisms

Inflammation acts as the critical “second hit”, which accelerates and perpetuates the vascular remodeling described above. PAH is increasingly recognized as a disease deeply intertwined with immune dysregulation, where both innate and adaptive immune cells are activated and infiltrate the pulmonary vasculature. Innate immune cells, such as macrophages, dendritic cells (DCs), mast cells, and neutrophils, accumulate around the pulmonary arteries and orchestrate a direct crosstalk with vascular cells. For example, macrophages, which are the predominant inflammatory cells in PAH lesions, polarize into M1 and M2 phenotypes, secreting chemokines like CX3CR1 and growth factors such as PDGF. This macrophage-derived PDGF directly stimulates PASMC proliferation, mechanistically linking immune activation to medial hypertrophy [19]. DCs, particularly monocyte-derived subsets, have been shown to influence PASMC proliferation and suppress endothelial gene expression, altering vascular pathology in both idiopathic and hereditary PAH [20].

The activation of innate immune sensors such as interferon-inducible protein 16 (IFI16) in PAECs induces proinflammatory cytokine production and apoptosis, mediated by epigenetic modifications such as m6A methylation [21]. This highlights how endothelial injury and immune activation form a vicious feed-forward loop. Adaptive immunity also contributes significantly to this process, with increased infiltration of T and B lymphocytes around remodeled vessels and elevated circulating autoantibodies, especially in connective tissue disease-associated PAH (CTD-PAH) [22,23]. Transcriptomic analyses have identified dysregulated B-cell receptor signaling pathways and humoral immune responses in experimental PAH models [24]. Chemokines, particularly CC chemokines, orchestrate immune cell recruitment, while platelets form aggregates with leukocytes and release pro-inflammatory mediators, linking thrombosis and immune activation during hypoxia [25,26,27]. Emerging evidence also implicates the gut microbiome as a novel modulator of systemic immunity in PAH, in which dysbiosis may facilitate disease progression [28,29].

Pharmacological validation of this inflammatory-proliferative axis is gaining traction. For instance, the kinase inhibitor seralutinib can interrupt macrophage-PASMC crosstalk [30]. Similarly, anti-inflammatory agents like interleukin-13 (IL-13) neutralizing antibodies have demonstrated preclinical efficacy by attenuating type 2 inflammation [31]. Furthermore, immunotherapies targeting NF-κB signaling or modulating macrophage polarization via mesenchymal stromal cell-derived exosomes also show potential [32,33]. However, a critical knowledge gap remains: while targeted immunotherapies succeed in preclinical rodent models, their translation to human clinical trials often yields limited efficacy, likely due to the extreme redundancy of human immune pathways and the late-stage, irreversible nature of established vascular lesions [34].

### 2.3 Genetics and Molecular Mechanisms

The structural and inflammatory changes in PAH are fundamentally rooted in genetic and epigenetic susceptibilities, which serve as the “soil” for disease initiation. Among the most critical genetic factors are mutations in the bone morphogenetic protein receptor type II (*BMPR2*) gene, encoding the bone morphogenetic protein receptor type II (BMPR2), a receptor integral to the transforming growth factor-beta (TGF-β) superfamily signaling pathway. *BMPR2* mutations are the most common genetic cause of heritable PAH (approximately 70–80% of familial cases and 10–20% of idiopathic PAH cases). These mutations lead to loss of function or haploinsufficiency, tipping the balance of vascular homeostasis towards PASMC proliferation and apoptosis resistance [35,36,37]. Expanding beyond classic cohorts, novel *BMPR2* mutations have been identified across diverse populations, including pediatric patients and those with no family history, underscoring the broad genetic heterogeneity [38,39]. Critically, the incomplete penetrance of *BMPR2*-related PAH remains a central clinical paradox, strongly supporting the “two-hit hypothesis” wherein this genetic vulnerability requires a secondary trigger, such as the inflammatory dysregulation or hypoxic insults discussed earlier, to manifest as clinical PAH.

Beyond *BMPR2*, other gene variants contribute to PAH genetic susceptibility. *NOTCH3*, encoding a transmembrane receptor (NOTCH3), has emerged as a significant player in PAH pathobiology. Gain-of-function mutations in *NOTCH3* enhance oxidative and endoplasmic reticulum (ER) stress pathways, leading to increased PASMC contraction and vascular dysfunction. Experimental models with *NOTCH3* mutations demonstrate amplified pulmonary hypertension phenotypes, mediated through downstream effectors such as Hes5 and Rho-associated kinase (ROCK) activation, highlighting the intricate molecular interplay between NOTCH3 signaling and vascular remodeling [40]. In specific disease contexts like congenital heart disease-associated PAH (APAH-CHD) and sarcoidosis-associated PH, variants in genes including *BMPR2*, *ACVRL1*, and *EIF2AK4* have been identified, suggesting that genetic predisposition modulates disease severity and progression in these secondary forms of PAH [41]. Similarly, mutations in *TBX4* disrupt lung morphogenesis and PAEC function through impaired fibroblast growth factor 10 (FGF10) signaling [42,43]. Beyond traditional genetics, the role of clonal hematopoiesis and epigenetic regulators such as DNA methyltransferases (DNMTs) has gained attention in PAH. Somatic mutations in *DNMT3A*, a key enzyme in DNA methylation, have been linked to increased inflammation and vascular remodeling, suggesting an epigenetic layer of regulation in PAH pathogenesis [44].

The pathological dominance of this genetic signaling imbalance is clinically validated by the advent of sotatercept, a novel activin signaling inhibitor that restores homeostasis between the hyperactive Activin/SMAD2/3 axis and the deficient BMP/SMAD1/5/8 pathway [45]. Ultimately, the profound vascular remodeling characteristic of PAH emerges from this complex crosstalk between aberrant genetic signaling, endothelial injury, and immune dysregulation. A multi-hit pathogenic cascade is delineated in Fig. 1. Understanding these genetic and molecular mechanisms not only elucidates disease etiology, but also informs the development of precision medicine approaches, including genetic counseling, molecular diagnostics, and targeted therapeutics for PH patients [35,46,47].

**Fig. 1.**
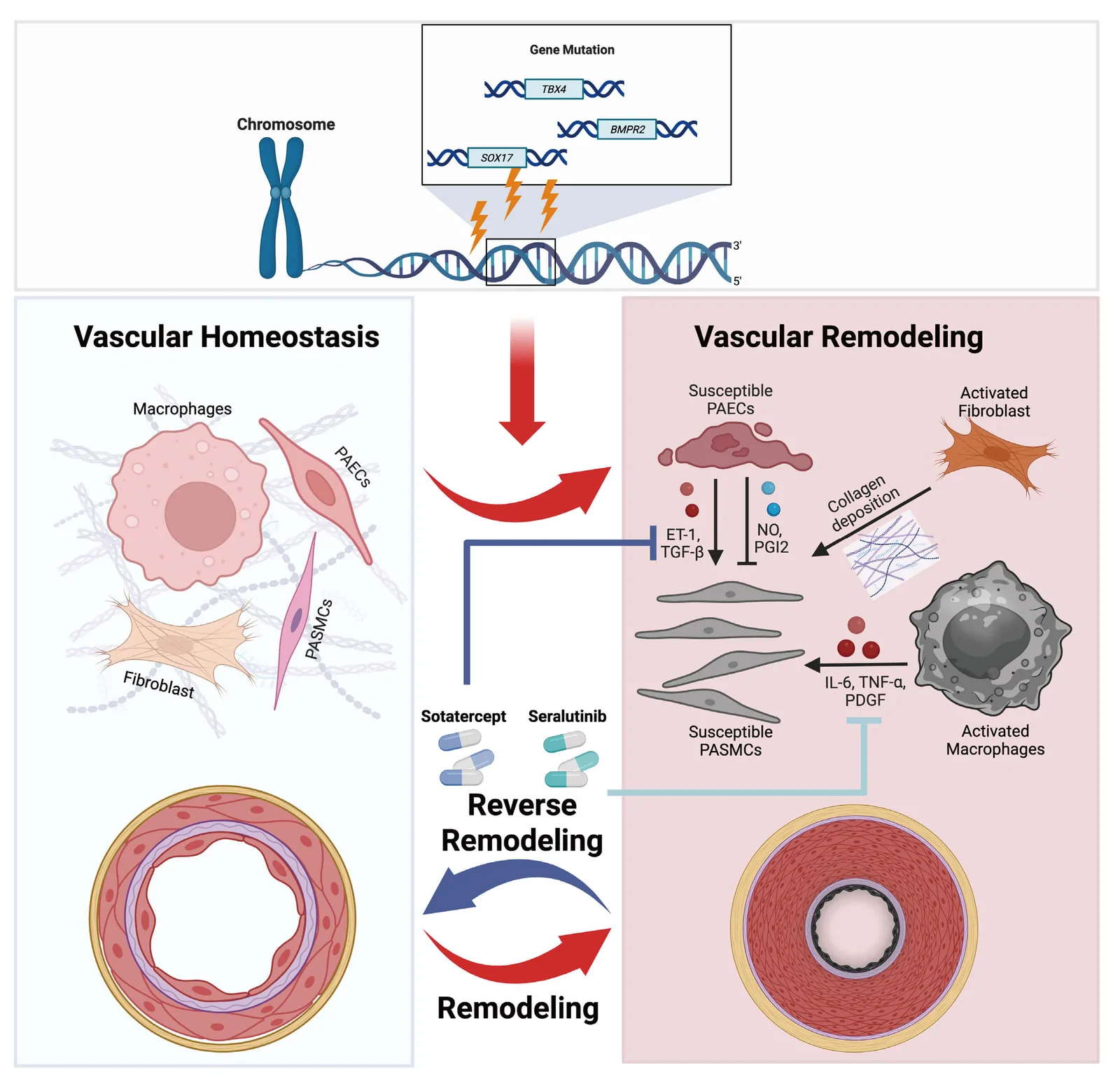
**Schematic representation of pulmonary vascular remodeling and therapeutic interception**. Genetic predispositions, including mutations in *BMPR2*, *SOX17*, and *TBX4*, trigger the transition from vascular homeostasis to pathogenic remodeling. Dysfunctional pulmonary arterial endothelial cells (PAECs) exhibit an imbalance of vasoactive mediators, driving pulmonary arterial smooth muscle cell (PASMC) proliferation. Concurrently, activated macrophages secrete pro-inflammatory cytokines (IL-6, TNF-α, PDGF), and activated fibroblasts drive collagen deposition, culminating in vessel occlusion. Novel disease-modifying agents, such as sotatercept and seralutinib, target these aberrant signaling cascades to promote reverse remodeling and restore vascular homeostasis. Created in BioRender.

Importantly, the heterogeneous pathobiological mechanisms discussed above influence the distinct clinical subgroups of PH, fundamentally dictating the WHO classification and tailored management strategies. For instance, while profound microvascular remodeling driven by genetic predispositions and endothelial dysfunction predominantly defines Group 1 PAH, unresolved thromboembolic fibrosis and distinct inflammatory cascades are the hallmarks of Group 4 CTEPH. In contrast, Group 2 and Group 3 PH are primarily driven by left heart filling pressures and hypoxia-induced vasoconstriction, respectively. Understanding these divergent predominant pathways is crucial for classification and class-specific therapies.

## 3. Clinical Classification and Etiological Analysis of Pulmonary Hypertension

### 3.1 Pulmonary Arterial Hypertension (PAH)

PAH is a rare but severe disease characterized by progressive elevation of pulmonary artery pressure, leading to right ventricular failure and death if untreated. Epidemiological data indicate an estimated prevalence of approximately 10.6 cases per million adults in the United States, with substantial morbidity and mortality [48]. Idiopathic PAH (IPAH) and heritable PAH represent primary forms of the disease. While IPAH is diagnosed in the absence of identifiable causes, heritable PAH is intimately linked to genetic mutations, predominantly within the *BMPR2* and *SOX17* genes. These mutations disrupt TGF-β and BMP pathways, promoting PASMC proliferation and endothelial dysfunction. IPAH typically presents with exertional dyspnea and fatigue, with the diagnosis confirmed via RHC demonstrating pre-capillary pulmonary hypertension [49,50]. Unlike IPAH, connective tissue disease-associated PAH (CTD-PAH), particularly systemic sclerosis-associated PAH (SSc-PAH), is a secondary manifestation driven by autoimmune mechanisms and chronic inflammation. Clinically, SSc-PAH patients exhibit distinct phenotypic traits, including a higher prevalence of positive anticentromere antibodies, older age at diagnosis, and prolonged disease duration [51]. Despite presenting with ostensibly better hemodynamic profiles than IPAH cohorts, CTD-PAH patients often suffer from limited exercise capacity and poorer prognosis [52]. This discrepancy may reflect greater peripheral muscle dysfunction and impaired ventilation, as evidenced by cardiopulmonary exercise testing [53]. Overlap with the underlying connective tissue disease often delays early diagnosis, prompting the adaptation of specialized risk stratification tools to assess prognosis and guide therapy in SLE-associated PAH and other CTD-PAH subtypes [54].

The therapeutic management of PAH has significantly evolved, targeting three major pathways: endothelin receptor antagonists (ERAs), phosphodiesterase type 5 inhibitors (PDE5i), and prostacyclin analogues. Calcium channel blockers remain effective in only a small subset of vaso-reactive IPAH patients. The treatment paradigm has shifted from sequential monotherapy to early combination regimens, a strategy shown to improve both morbidity and mortality. As a result, upfront dual therapy with ERAs and PDE5i is now the standard of care for most patients without significant comorbidities, whereas triple therapy, incorporating prostacyclin analogues, is reserved for high-risk cases [55,56,57]. Emerging therapies are beginning to target vascular remodeling rather than vasodilation alone. The activin signaling inhibitor sotatercept restores BMP/TGF-β homeostasis, while the inhaled kinase inhibitor seralutinib suppresses PDGFR and CSF1R-driven proliferation [30,45,58,59]. Parallel innovations in delivery, such as inhaled prostacyclin formulations such as Yutrepia, have demonstrated favorable safety and tolerability, offering convenient routes of administration [60].

The distinct therapeutic responses observed in CTD-PAH patients necessitate highly tailored management approaches. For example, immunomodulatory treatments including rituximab and dimethyl fumarate are currently under investigation for SSc-PAH, reflecting the fundamental autoimmune component of the disease [61]. Building upon this targeted approach, risk assessment models adapted for CTD-PAH and SLE-associated PAH have been instrumental in predicting outcomes and guiding treatment intensification [54]. Ultimately, the prognosis of CTD-PAH remains inferior to IPAH despite better hemodynamics, a reality largely driven by the increased systemic disease burden and associated comorbidities [52,53].

### 3.2 Pulmonary Hypertension Associated With Left Heart Disease (PH-LHD)

PH associated with left heart disease (PH-LHD) is the most common form of PH worldwide, predominantly driven by elevated left atrial pressure resulting from left heart dysfunction. PH-LHD results from elevated left-sided filling pressures that are transmitted retrograde to the pulmonary circulation, leading to isolated post-capillary pulmonary hypertension (IpcPH) [62,63]. Initially, this congestion manifests as passive pressure transmission without intrinsic pulmonary vascular remodeling. Over time, however, sustained left atrial hypertension in a subset of patients triggers pulmonary vascular remodeling. This remodeling is characterized by intimal fibrosis, medial hypertrophy, and adventitial changes in pulmonary arteries, leading to increased PVR and a combined pre- and post-capillary PH (CpcPH) phenotype [63,64,65], with involvement of key vaso-regulatory pathways including nitric oxide, endothelin signaling, and MAP kinase cascades [66,67]. As passive pulmonary venous hypertension evolves into vascular remodeling, right ventricular (RV) function progressively deteriorates due to increasing afterload. This cascade culminates in right ventricular–pulmonary artery (RV-PA) uncoupling and right heart failure, predicting a poorer prognosis [63,68].

The pathophysiology of PH-LHD is further complicated by atrial functional impairment and left atrial enlargement, which correlate directly with elevated pulmonary pressures and vascular resistance [69,70]. Strain imaging can unmask early left atrial dysfunction and elevated pulmonary capillary wedge pressure early in the course of the disease, even during exercise, offering a critical window for early detection [69]. In addition, systemic comorbidities ranging from hypertension and atrial fibrillation to obesity and metabolic syndrome can exacerbate the progression of PH-LHD [64,65].

Diagnostic differentiation between IpcPH and CpcPH is crucial, as the latter correlates with worse outcomes and distinct management requirements. Hemodynamic definitions have evolved, with the 2022 ESC/ERS guidelines emphasizing the use of a strict PVR threshold (>2 Wood Units) to accurately stratify these subtypes [2,71]. This progression from passive congestion to vascular remodeling and right ventricular failure highlights the complexity of PH-LHD.

Management of PH-LHD fundamentally centers on optimizing the primary cardiac pathology, given that standard PAH-approved pulmonary vasodilators have failed to demonstrate consistent efficacy and may cause harm in this population [63,72]. Current guidelines strongly recommend addressing the underlying left heart disease, whether heart failure with reduced ejection fraction (HFrEF), heart failure with preserved ejection fraction (HFpEF), or valvular heart disease, as the basis for therapeutic interventions [73,74]. Pharmacologic interventions rely on guideline-directed medical therapy for heart failure, deploying angiotensin-converting enzyme inhibitors, beta-blockers, mineralocorticoid receptor antagonists, and diuretics to alleviate left ventricular filling pressures and mitigate pulmonary venous congestion [64,75]. For structural etiologies such as valvular heart disease, timely surgical or percutaneous interventions correcting mitral or aortic valve pathology effectively decompress left atrial pressures and restore pulmonary hemodynamics [75]. Rigorous control of comorbidities is equally essential to optimize cardiac function and reduce the progression of PH-LHD [64,76].

While advanced therapies targeting the pulmonary vasculature (PDE5i, ERAs, and prostacyclin analogues) have undergone evaluation in clinical trials, they have yielded no definitive benefit and may increase adverse events. Consequently, their routine use outside of clinical trials is not currently recommended [72,77,78]. Although some pediatric PH specialists may occasionally use pulmonary vasodilators in complex CpcPH cases presenting with right ventricular dysfunction, there is an absence of broad consensus on the use of these drugs, and practice patterns vary significantly [78]. In summary, PH-LHD management remains firmly anchored in treating the underlying left heart disease, and relegating the use of pulmonary vasodilators to an investigational role requiring cautious application at specialized centers.

### 3.3 Pulmonary Disease-Associated Pulmonary Hypertension

PH associated with pulmonary diseases (Group 3 PH) encompasses a heterogeneous spectrum of disorders, primarily chronic obstructive pulmonary disease (COPD) and interstitial lung diseases (ILDs). The pathophysiology is multifactorial, involving hypoxic pulmonary vasoconstriction, vascular remodeling, and progressive destruction of the pulmonary vascular bed secondary to parenchymal damage. In COPD and ILD, chronic hypoxia induces sustained vasoconstriction, increasing PVR and resulting in subsequent right ventricular strain. Intrinsic alterations, including intimal fibrosis, medial hypertrophy, and adventitial remodeling, contribute significantly to the disease progression [79]. Diagnosing PH within the context of chronic pulmonary diseases presents significant challenges due to overlapping clinical and radiographic features, where severe parenchymal abnormalities can obscure standard hemodynamic assessments. While RHC remains the gold standard, accurate interpretation requires meticulous correlation with lung function and imaging studies to differentiate PAH from PH secondary to lung disease.

Treatment strategies for Group 3 PH are similarly complex. While targeted PAH therapies have revolutionized Group 1 management, their efficacy in Group 3 PH remains highly ambiguous. PAH-targeted therapies may improve hemodynamics but generally fail to improve exercise capacity in patients with severe chronic lung diseases associated with PH [80,81]. Moreover, concerns about worsening ventilation-perfusion mismatch and hypoxemia have limited the widespread use of these agents. Addressing this limitation, the INCREASE trial demonstrated that inhaled treprostinil improves exercise capacity and lowers NT-proBNP levels in patients with PH-ILD [82]. By delivering the vasodilator directly to well-ventilated lung units, the inhaled route minimizes ventilation-perfusion mismatch. Subsequent extension and post-hoc analyses further confirmed its long-term safety, sustained functional benefits, and efficacy in reducing clinical worsening [83,84]. In systemic sclerosis-associated PH with concomitant ILD, PAH-specific therapies yield hemodynamic improvements but limited functional benefit, with the mere presence of ILD signaling a worse prognosis [85,86]. A notable exception in this challenging landscape is the emerging utility of sotatercept, which has shown potential in selected patients with CTD-PAH and coexisting ILD, although evidence remains limited [87]. For patients reaching end-stage disease, lung transplantation remains the definitive option, though it continues to be constrained by complex timing and rigorous patient selection criteria [88,89]. Overall, Group 3 PH is primarily driven by parenchymal lung disease, which limits the role of PAH-specific therapies, although inhaled prostacyclin therapy has emerged as a targeted option.

### 3.4 Chronic Thromboembolic Pulmonary Hypertension (CTEPH)

CTEPH is a distinct and potentially curable form of pulmonary hypertension, defined by persistent obstruction of pulmonary arteries by organized thromboembolic material. This chronic obstruction precipitates progressive pulmonary vascular remodeling, elevated PVR, and ultimate right heart failure. The core pathophysiology hinges on unresolved thromboemboli that undergo fibrotic transformation, adhering to the pulmonary arterial wall. This proximal obstruction initiates a secondary microvasculopathy, driving remodeling of the distal small pulmonary arteries and capillary beds. This dual process underlies the hemodynamic abnormalities in CTEPH. Radiological studies have revealed that while both chronic thromboembolic pulmonary disease (CTEPD) and CTEPH may exhibit similar macroscopic thrombotic burdens, CTEPH patients exhibit a more extensive microvascular disease profile, characterized by larger perfusion defects and mosaic attenuation patterns on imaging [90]. CT pulmonary angiography (CTPA) and dual-energy CT have emerged as valuable tools not only for detecting vascular obstructions, but also for assessing pulmonary perfusion heterogeneity, which correlates with disease severity and residual post-interventional PH [91,92,93].

The clinical presentation of CTEPH is nonspecific, dominated by exertional dyspnea and fatigue that frequently delays diagnosis. A history of acute pulmonary embolism is present in approximately 65% of patients, but CTEPH can also develop without a documented embolic event, underscoring the need for high clinical suspicion and systematic screening in patients with unexplained pulmonary hypertension [94,95]. Beyond mere mechanical obstruction, the pathogenesis is modulated by inflammatory and genetic variables. Recent studies have mapped heterogeneous genetic variants related to coagulation, fibrinolysis, and vascular remodeling pathways, suggesting a complex, multi-hit mechanism driving the development of CTEPH [96,97]. To facilitate earlier detection, novel biomarkers such as soluble ST2 have shown predictive value in identifying patients at high risk of transitioning from acute pulmonary embolism to CTEPH, offering a critical tool for early risk stratification [98].

Pulmonary endarterectomy (PEA) remains the gold standard and the only potentially curative treatment for operable CTEPH, structurally removing the organized thromboembolic material from the pulmonary arterial tree [99]. Operability is determined by the location and accessibility of thromboembolic lesions, the patients’ functional status, and underlying comorbidities. However, approximately 30–40% of patients are deemed inoperable due to the distal distribution of the disease or the presence of severe comorbidities [94,100]. For these patients, balloon pulmonary angioplasty (BPA) has emerged as an effective interventional alternative. BPA involves percutaneous catheter-based dilation of obstructed pulmonary arteries, improving pulmonary hemodynamics and exercise capacity with an acceptable safety profile [100,101,102,103]. Recent studies have demonstrated that functional assessment-guided BPA, using pressure measurements to optimize dilation endpoints, can enhance treatment efficacy and reduce complications, suggesting that a tailored approach to BPA may improve outcomes [103]. The contemporary management of CTEPH has evolved into a multimodal paradigm. This framework integrates PEA, BPA, and targeted medical therapy such as the soluble guanylate cyclase stimulator riociguat to achieve optimal pulmonary revascularization [94,104,105]. For highly selected high-risk CTEPH patients, a multimodal approach integrating PEA and BPA offers a new therapeutic option [106]. For patients presenting with mixed proximal and distal thromboembolic disease, a sequential hybrid strategy comprising initial PEA followed by BPA is increasingly implemented to address anatomically diverse lesions [107]. Across all therapeutic modalities, lifelong anticoagulation remains a cornerstone of management to prevent recurrent thrombosis, though debate persists regarding the comparative efficacy of vitamin K antagonists versus direct oral anticoagulants in this specific population [108,109]. Despite these advances, residual post-operative pulmonary hypertension afflicts approximately one-quarter of PEA recipients, resulting in a significantly worse prognosis [110]. The presence of residual PH necessitates long-term surveillance and may require additional interventions with BPA or medical therapy to stabilize hemodynamics and obtain long-term clinical stability.

### 3.5 Pulmonary Hypertension With Unclear and/or Multifactorial Mechanisms

PH with unclear and/or multifactorial mechanisms (Group 5 PH) encompasses a heterogeneous set of conditions in which PH arises from unclear or multifactorial mechanisms, often involving a combination of hematologic, systemic, metabolic, and other rare disorders. Representative conditions include sarcoidosis, chronic kidney disease, myeloproliferative disorders, and metabolic diseases such as glycogen storage disorders [111,112]. The underlying mechanisms are highly variable and may include extrinsic vascular compression, intrinsic vascular remodeling, inflammatory infiltration, or high-output states, frequently coexisting within the same patient [111]. Given this heterogeneity, management is primarily directed toward the underlying disease, and evidence supporting PAH-targeted therapies remains limited and inconsistent.

## 4. Diagnostic Techniques and Risk Assessment of Pulmonary Hypertension

### 4.1 Imaging and Functional Assessment

As illustrated in Fig. 2, the clinical evaluation of PH requires a comprehensive step-wise algorithm [2]. Echocardiography, particularly transthoracic echocardiography (TTE), serves as the non-invasive method for initial screening and ongoing risk stratification of PH. Its widespread availability, noninvasive nature, and ability to provide real-time hemodynamic and functional information make it indispensable in clinical practice. TTE primarily detects RV pressure overload and dysfunction, which are critical markers of disease severity and prognosis in PAH. Parameters including tricuspid annular plane systolic excursion (TAPSE), RV fractional area change (FAC), and the ratio of TAPSE to pulmonary artery systolic pressure (PASP) have emerged as important indicators of RV-PA coupling, which significantly correlates with exercise capacity and long-term survival [113,114]. However, it is crucial to note in clinical practice that relevant PH is invariably associated with increasing secondary tricuspid regurgitation (TR). As TR progresses alongside the aggravation of RV dilation, parameters such as TAPSE and FAC become increasingly unreliable and can even be misleading regarding true right ventricular function. Advanced techniques such as speckle tracking and tissue Doppler imaging enable detailed evaluation of RV strain and deformation, providing early detection of RV dysfunction before overt clinical deterioration occurs [115]. Consequently, contemporary risk stratification algorithms increasingly integrate these dynamic functional indices to better predict mortality and tailor targeted therapies [114]. Similar principles apply to pediatric cohorts, where specialized protocols have been developed to accommodate the unique developmental trajectory of the juvenile right heart [116].

**Fig. 2.**
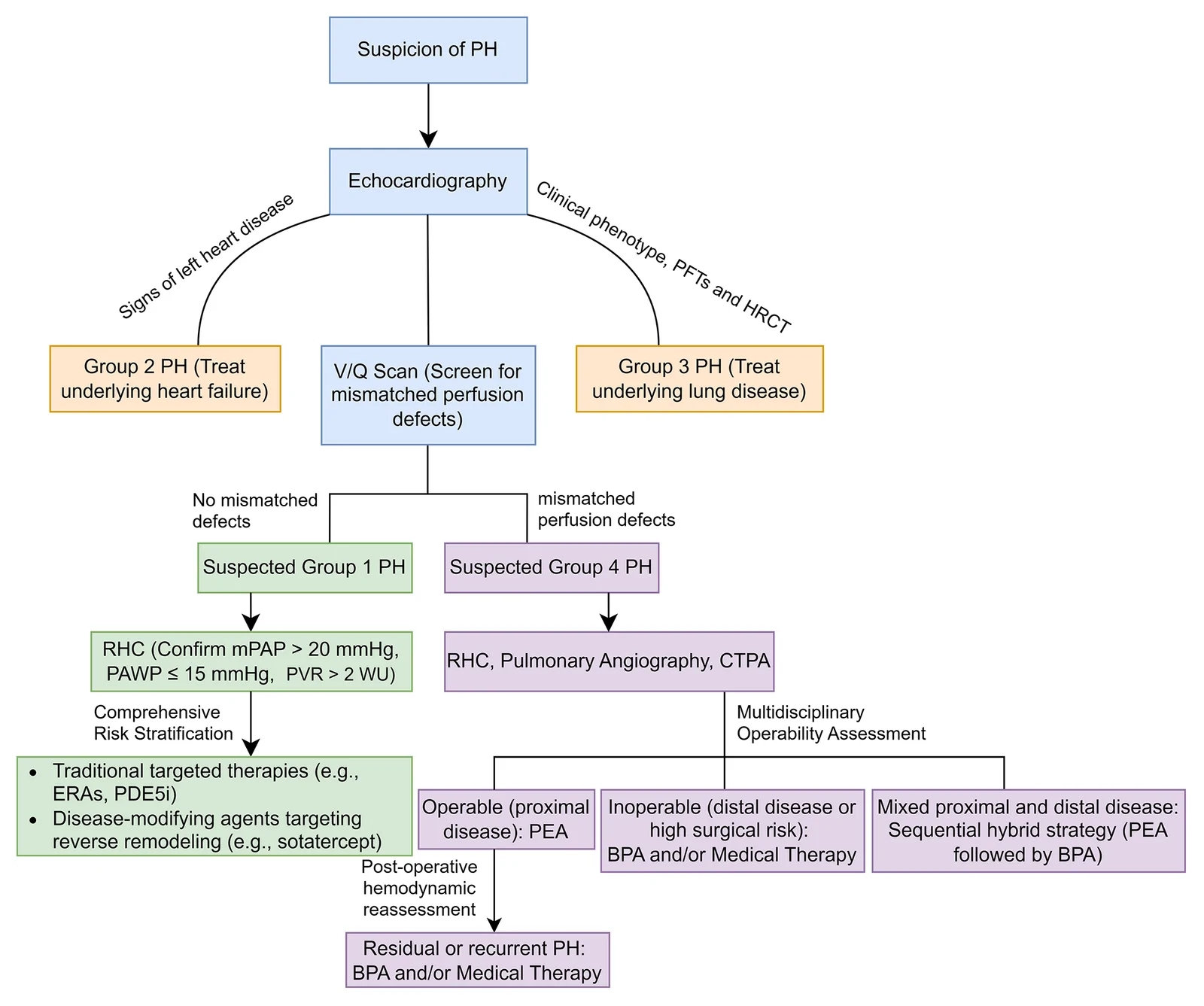
**Diagnostic and therapeutic algorithm for pulmonary hypertension**. PH, pulmonary hypertension; PFTs, pulmonary function tests; HRCT, high-resolution computed tomography; V/Q, ventilation/perfusion; RHC, right heart catheterization; mPAP, mean pulmonary artery pressure; PVR, pulmonary vascular resistance; WU, Wood units; ERAs, endothelin receptor antagonists; PDE5i, phosphodiesterase type 5 inhibitors; CTPA, computed tomography pulmonary angiography; PEA, pulmonary endarterectomy; BPA, balloon pulmonary angioplasty; PAWP, pulmonary artery wedge pressure.

To resolve complex anatomical and functional phenotypes, cardiac magnetic resonance imaging (CMR) and CTPA serve as indispensable complementary modalities. CMR is considered the gold standard for noninvasive assessment of RV morphology and function due to its high spatial resolution, reproducibility, and ability to quantify ventricular volumes, mass, and ejection fraction without using ionizing radiation [117,118,119]. More importantly, phase-contrast imaging enables evaluation of pulmonary artery flow dynamics and stiffness, which are important in understanding pulmonary vascular load and RV-PA coupling [120]. Tissue-level characterization via CMR further identifies myocardial fibrosis and edema, which have prognostic implications in PAH [121,122]. CTPA offers high-resolution visualization of the pulmonary vasculature, enabling detection of vascular remodeling, thromboembolic lesions, and arterial dilation characteristic of PAH and CTEPH [123,124]. Quantitative CT metrics, such as three-dimensional volume measurements of the main and branch pulmonary arteries, correlate well with invasive hemodynamics and strengthen diagnostic confidence [123]. Advanced CT techniques, including dual-energy CT and artificial intelligence-based analyses, allow for the precise differentiation of arterial versus venous involvement, assessment of vascular pruning and tortuosity, and early detection of disease phenotypes [125]. Additionally, novel molecular imaging approaches using PET tracers targeting fibroblast activation protein or TGF-β receptors provide insights into pulmonary vascular remodeling and inflammation in PAH [126,127]. The integration of CMR and CT data enhances diagnostic precision, facilitates differentiation between PH subtypes, and guides therapeutic strategies including PEA and BPA in CTEPH patients [128,129].

### 4.2 Biomarkers and Immunological Indicators

The clinical utility of biomarkers such as N-terminal pro-brain natriuretic peptide (NT-proBNP), inflammatory cytokines, and immune cell indicators has been extensively studied in PH, offering valuable insights into disease severity, prognosis, and therapeutic monitoring. NT-proBNP, a cardiac neurohormone released in response to RV strain, remains the most widely used biomarker in clinical practice for assessing RV dysfunction and risk stratification in PAH patients. While elevated NT-proBNP levels correlate with increased pulmonary artery pressures and adverse outcomes, its diagnostic performance is generally considered moderate across clinical studies, with relatively high sensitivity but limited specificity, particularly in the presence of renal dysfunction or systemic comorbidities [130,131]. To overcome this limitation, recent studies have explored multi-dimensional protein panels. A newly identified three-biomarker panel comprising β-nerve growth factor (β-NGF), chemokine CXCL9, and tumor necrosis factor-related apoptosis-inducing ligand (TRAIL) independently outperforms traditional variables in predicting survival and clinical outcomes, demonstrating superior discriminative capacity in validation cohorts [131]. In addition, inflammatory mediators such as interleukin-6 (IL-6), IL-8, and chemokines CXCL9 and CXCL13 have been found to be elevated in specific PAH subgroups, reflecting the inflammatory milieu and correlating with disease progression and survival [132]. Immune cell profiling reveals alterations in circulating and tissue-resident immune populations, including increased M1 macrophages, activated DCs, and T cell subsets, which contribute to endothelial injury and vascular remodeling [133,134,135]. The expression of immune checkpoint molecules such as CD200 and its receptor CD200R on T lymphocytes has been linked to IPAH, suggesting their role as non-invasive immune markers associated with disease pathogenesis and EBV reactivation [136]. In addition, microRNAs like *miR-17* have emerged as potent diagnostic and prognostic biomarkers, correlating with functional parameters such as 6-minute walk distance and pulmonary artery pressures [137]. Crucially, autoantibodies (AABs) such as those against α1-adrenergic and endothelin-1A receptors (α1-ARs and ETARs, respectively) are particularly involved in IPAH. Earlier studies revealed that the majority of PAH patients have AABs against both α1-ARs and ETARs [138]. The integration of multiple biomarkers, including NT-proBNP, inflammatory cytokines, and immune cell markers, into composite risk models has improved the ability to predict mortality in this group of patients and may facilitate personalized therapeutic decision-making [139].

Emerging immunological biomarkers hold promise for enhancing early diagnosis and dynamic monitoring of therapeutic efficacy in PAH. Proteomic interrogation has revealed upregulation of galectin-3 binding protein (LGALS3BP), apelin-17, and ectodysplasin A2 receptor (EDA2R), which demonstrate high sensitivity in preliminary proteomic studies for distinguishing IPAH and CTD-PAH [140,141]. Machine learning approaches integrating multi-omics data have identified gene panels including *CALD1*, *SLC7A11*, and immune-related long non-coding RNAs (lncRNAs) such as *JPX-RABEP1*. These genetic signatures directly correlate with immune cell infiltration, offering both mechanistic insights and diagnostic value [142,143]. Collectively, these biomarkers reflect different stages of clinical translation, ranging from established markers used in routine risk stratification (e.g., NT-proBNP) to emerging candidates that require further validation in large-scale cohorts. Moving from correlation to intervention, immunomodulatory cytokines such as IL-6 have been implicated as both biomarkers of disease severity and therapeutic targets, with clinical trials underway to evaluate anti-IL-6 receptor antibodies in immune-responsive PAH phenotypes [132]. In the expanding pathogenic landscape, the gut-lung axis and microbiota-derived metabolites are now recognized as contributors to immune dysregulation in PAH [144]. Despite these advances, challenges remain in standardizing assays, validating biomarkers across diverse cohorts, and integrating multi-dimensional data into clinically actionable tools. Future research focusing on longitudinal validation and combinatorial biomarker panels is essential to realize the full potential of immunological markers in early detection and real-time monitoring of the efficacy of these treatments.

## 5. Existing Treatment Strategies of Pulmonary Hypertension

### 5.1 Drug Therapy

The pharmacological landscape of PAH is evolving from traditional vasodilatory regimens toward emerging disease-modifying therapies targeting vascular proliferation (Table 1). For decades, the pharmacological management of PAH has been shaped by targeting three classical dysfunctional pathways implicated in endothelial dysfunction: the endothelin pathway, the nitric oxide (NO) pathway, and the prostacyclin pathway. ERAs such as bosentan, ambrisentan, and macitentan counteract the potent vasoconstrictive and proliferative effects of endothelin-1 by blocking its receptors, thereby reducing PVR and improving symptoms. PDE5i, like sildenafil and tadalafil, enhance the NO pathway by preventing the degradation of cyclic guanosine monophosphate (cGMP), thereby promoting vasodilation and exerting antiproliferative effects on PASMCs. Prostacyclin analogues and receptor agonists, including epoprostenol, treprostinil, and selexipag, mimic the effects of endogenous prostacyclin, leading to vasodilation and inhibition of platelet aggregation. While these conventional agents collectively alleviate symptoms and improve hemodynamics, they primarily act as vasodilators rather than reversing vascular remodeling or fundamentally altering disease progression [145,146,147].

**Table 1.** **Summary of Established and Emerging Pharmacological Therapies for Pulmonary Arterial Hypertension**.

Drug Class	Target/Mechanism of Action	Representative Agents	Current Development Status
Endothelin Receptor Antagonists (ERAs)	Block ET-1 binding to ETA/ETB receptors, reducing vasoconstriction and PASMC proliferation.	bosentan, ambrisentan, macitentan	Approved/Standard of care (foundation of combination therapy)
Phosphodiesterase type 5 Inhibitors (PDE5i)	Inhibit PDE5, preventing cGMP degradation to enhance NO-mediated vasodilation.	sildenafil, tadalafil	Approved/Standard of care
Soluble Guanylate Cyclase (sGC) Stimulators	Directly stimulate sGC independently of NO, increasing intracellular cGMP levels.	riociguat	Approved for PAH and inoperable/residual CTEPH after PEA
Prostacyclin Pathway Agonists	Bind to IP receptors, mimicking endogenous PGI2 to induce vasodilation and anti-proliferation.	epoprostenol, treprostinil, selexipag	Approved/Standard of care (essential for high-risk patients)
Activin Signaling Inhibitors	Fusion protein acting as a ligand trap for TGF-β superfamily members (e.g., activins), restoring signaling balance.	sotatercept	Approved (FDA 2024) based on the Phase III trial
Inhaled Tyrosine Kinase Inhibitors (TKIs)	Inhibit multiple kinases including PDGFR, CSF1R, and c-KIT, targeting local inflammation and proliferation.	seralutinib	Phase III (PROSERA study ongoing) following Phase II validation

Abbreviations: PAH, pulmonary arterial hypertension; CTEPH, chronic thromboembolic pulmonary hypertension; ET-1, endothelin-1; cGMP, cyclic guanosine monophosphate; NO, nitric oxide; PASMC, pulmonary artery smooth muscle cell; PEA, pulmonary endarterectomy; TGF-β, transforming growth factor-beta.

To address the underlying autoimmune mechanisms, non-pharmacological approaches have also been explored. Notably, in patients with IPAH, the removal of these cardiac autoantibodies by immunoadsorption (IA) therapy has already confirmed benefits, providing a valuable therapeutic option [138,148]. This approach is further supported by established evidence in dilated cardiomyopathy (DCM), where the prevalence of functional β1-adrenergic receptor autoantibodies (β1AR-AAbs) positively correlates with heart failure severity. Clinical responses to IA therapy in these patients, such as a notable reduction in pulmonary vascular resistance, indicate that this intervention effectively disrupts the vicious cycle between cardiac inflammation and β1AR-AAb production [149].

Recognizing the pathological complexity of PAH, the field has transitioned from sequential add-on strategies to upfront combination therapies. Clinical trials have demonstrated that initial combination therapy, such as ambrisentan with tadalafil, yields superior improvements in exercise capacity and delays disease progression compared to monotherapy. Despite these survival benefits, a critical knowledge gap persists regarding the precise intracellular crosstalk between these three classical pathways and how their simultaneous modulation affects downstream right ventricular adaptation. In the real-world clinical setting, implementation of these treatments faces hurdles, including drug-drug interactions, particularly those mediated by cytochrome P450 enzymes when combining ERAs with oral prostacyclin agents. Clinicians must remain vigilant to these interactions to ensure patient safety [150,151].

To address the pressing unmet need for disease-modifying interventions, contemporary research has pivoted toward pathways capable of reversing structural pulmonary vascular remodeling. Novel agents focus on pathways such as the bone morphogenetic protein (BMP)/transforming growth factor-beta (TGF-β) signaling axis, which is dysregulated in PAH. A breakthrough in this domain is sotatercept, a novel activin receptor ligand trap recently validated in the STELLAR trial, which acts to restore homeostasis between growth-promoting and inhibitory signaling, thereby directly targeting proliferative vascular remodeling [45]. It has demonstrated significant clinical benefit in a phase III trial, including reductions in pulmonary vascular resistance and improvements in exercise capacity, and has more recently been shown to reduce major morbidity and mortality events in high-risk patients in the ZENITH trial, further supporting a potential role in modifying disease progression [152]. Furthermore, the recently published HYPERION trial provided compelling evidence that early initiation of sotatercept in PAH patients diagnosed within 12 months substantially reduces the risk of clinical worsening [153]. Tyrosine kinase inhibitors (TKIs), such as imatinib, have demonstrated efficacy but are limited by systemic side effects; the inhaled kinase inhibitor seralutinib investigated in the TORREY trial offers targeted delivery with improved tolerability and improvement in 6-minute walk distance, which is currently in clinical development in a phase III study [30,154]. Additional approaches include anti-inflammatory therapies, metabolic modulators (e.g., ranolazine, sodium-glucose cotransporter 2 inhibitors), and hormonal modulation. In addition, drug repositioning strategies using network-based analyses have identified candidate drugs with potential efficacy in PAH by targeting underlying pathobiological mechanisms. Nanotherapeutic delivery systems are also being explored to enhance drug efficacy and reduce toxicity. Collectively, these approaches signal a critical transition toward precision medicine, aiming to improve long-term outcomes by addressing the complex molecular and cellular mechanisms involved in the progression of PAH [145,155,156,157,158,159,160].

Emerging therapies have introduced a mechanistic distinction from conventional treatments. Whereas established agents primarily act as vasodilators, sotatercept targets pulmonary vascular remodeling through modulation of activin-BMP signaling. While current evidence supports its use as an add-on therapy, its long-term impact on disease progression, potential to reverse established remodeling, and its role within combination regimens remain to be defined.

### 5.2 Interventional and Surgical Treatments

PEA remains the definitive and potentially curative intervention for CTEPH. Removing organized thromboembolic material from the pulmonary arterial tree, it restores pulmonary blood flow and reduces pulmonary artery pressure. Studies have demonstrated that PEA offers substantial hemodynamic improvement with low perioperative mortality when performed in experienced centers, leading to marked improvement in functional status and survival [161,162]. It also reduces right ventricular afterload and promotes reverse cardiac remodeling, with associated molecular changes including downregulation of inflammatory and profibrotic genes such as *EGR1* [163]. However, approximately 37% of patients with CTEPH are deemed inoperable due to the presence of distal lesions, comorbidities, advanced age, and residual or recurrent PH, which may occur after surgery [161]. Careful patient selection and management within specialized multidisciplinary teams are therefore essential to optimize outcomes in CTEPH patients.

BPA has emerged as an effective option for patients with inoperable CTEPH or persistent PH after PEA. BPA involves percutaneous catheter-based dilation of stenotic or occluded pulmonary artery branches, leading to improvements in hemodynamics and functional status. Recent refinements in BPA techniques have significantly enhanced safety, with reduced rates of reperfusion pulmonary edema compared with earlier experience [164]. Recent research utilizing cardiac magnetic resonance imaging demonstrates that while PEA achieves more pronounced hemodynamic unloading and right ventricular reverse remodeling, both interventions significantly improve cardiac function and exercise capacity [165]. This evidence solidifies BPA as a robust alternative for inoperable patients and validates its integration into sequential hybrid strategies and the management of residual pulmonary hypertension. Clinical experience from centers performing BPA demonstrates substantial reductions in mPAP and PVR, with five-year survival rates reaching approximately 90%, comparable to those of operable CTEPH patients undergoing PEA [164]. BPA improves pulmonary blood flow dynamics by decreasing blood flow stagnation and increasing wall shear stress, which promotes vasodilation and distal vessel remodeling. Safety data from large cohort studies reveal low rates of life-threatening complications, with most reperfusion injuries being subclinical and manageable [166]. BPA is particularly valuable for distal or subsegmental disease and is now considered a key component in the management of CTEPH. In selected cases, it may also be used to optimize hemodynamics prior to noncardiac surgery [167].

Distinct from established interventions for CTEPH, pulmonary artery denervation (PADN) offers a device-based approach for Group 1 PAH. By targeting perivascular sympathetic nerves, PADN mitigates vasoconstriction and vascular remodeling driven by sympathetic overactivity. Intravascular ablation improves pulmonary hemodynamics through consistent reductions in mPAP and PVR, alongside increased cardiac output [168,169]. Clinical research indicates that PADN enhances exercise capacity and decreases the incidence of clinical worsening over long-term follow-up, particularly when combined with targeted medical therapy [170]. Although its impact on mortality remains undetermined, PADN acts as a viable adjunctive intervention. Further randomized controlled trials comparing PADN with optimal medical therapy are warranted.

Lung transplantation remains the last-line option for patients with PAH who are refractory to pharmacological, interventional, and other surgical therapies. The perioperative management of lung transplantation in PAH patients is complex, requiring advanced support modalities such as veno-arterial extracorporeal membrane oxygenation (VA-ECMO) to stabilize hemodynamics and provide left ventricular conditioning [171]. However, right-to-left shunting procedures such as atrial septostomy or Potts shunt, used as bridging strategies, may have deleterious effects post-transplantation, necessitating careful consideration and possible closure of artificially created defects. The prognosis after lung transplantation depends on multiple factors, including preoperative status, perioperative management, and adherence to long-term immunosuppression. Given limited donor availability and substantial risk, transplantation is reserved for carefully selected patients and should be undertaken in experienced centers. Despite these challenges, it remains a life-saving option for end-stage disease.

### 5.3 Supportive Therapy and Comprehensive Management

Supportive therapy remains a cornerstone in the management of PH, particularly PAH, alongside targeted pharmacological treatments. Oxygen therapy is widely used to correct hypoxemia, thereby reducing pulmonary vasoconstriction, although the precise indicators and optimal oxygen saturation targets require individualized assessment, especially in patients with congenital heart disease or Eisenmenger syndrome, where shunting and cyanosis complicate management [172]. Diuretics are essential in managing right heart failure associated with PAH by reducing volume overload and systemic congestion. They improve symptoms but require careful titration to avoid excessive reduction of preload [2]. Anticoagulant therapy is often considered due to the prothrombotic milieu in PAH, particularly in idiopathic forms or those associated with CTEPH. Lifelong anticoagulation reduces the risk of *in situ* thrombosis and recurrent embolic events, which can worsen pulmonary vascular resistance and right ventricular function. However, its use should be individualized in light of the bleeding risk, especially in patients with comorbidities or those undergoing invasive procedures [173]. In specific etiologies such as pulmonary veno-occlusive disease, anticoagulation may be contraindicated due to the risk of pulmonary hemorrhage [174]. The perioperative use of pulmonary vasodilators and inodilators, including inhaled prostacyclin and nitric oxide, has demonstrated benefits in reducing pulmonary artery pressure and improving right ventricular function during cardiac surgery, although supporting evidence remains limited [175].

Psychological support is crucial given the chronic, progressive nature of PAH, which significantly impairs quality of life and imposes emotional burdens such as anxiety, depression, and social isolation. Studies highlight the importance of integrating mental health services, counseling, and patient education to improve coping strategies and adherence to therapy [176]. Lifestyle modifications, including tailored physical activity programs, nutritional optimization, and smoking cessation, contribute to better functional capacity and overall health. Exercise rehabilitation, when carefully supervised, can enhance exercise tolerance and reduce symptoms without precipitating right heart failure [177]. Nutritional support is particularly relevant in patients with congestive symptoms or those at risk of cachexia. Avoidance of high altitudes and prompt management of respiratory infections are also advised to prevent exacerbations.

The complexity of PH pathophysiology and its systemic impact underscore the need for multidisciplinary team (MDT) involvement. An integrated MDT approach enables accurate diagnosis, risk stratification, and individualized treatment, and is associated with improved clinical outcomes [178].

## 6. Conclusion

In conclusion, PH remains a complex and heterogeneous condition, driven by the intricate interplay of genetic predispositions, immune-inflammatory responses, and profound vascular remodeling. While our evolving pathophysiological understanding has transformed clinical management, shifting from palliative vasodilation to disease-modifying therapies (exemplified by sotatercept) and refining multidisciplinary interventions (including PEA and BPA for Group 4 CTEPH), significant hurdles remain. Moving forward, several priorities should be emphasized. First, translating multi-omics discoveries into clinical applications will require integration with longitudinal cohorts and functional validation to identify causal targets for drug therapy. Second, optimizing clinical trial design should adopt phenotype-enriched strategies and incorporate right ventricular function and RV–PA coupling as key endpoints. Third, advancing clinical translation will depend on scalable early detection and risk stratification approaches, integrating biomarkers, imaging, and prediction models to enable earlier and more precise intervention.
